# Inequalities in health-related quality of life and functional health of an aging population: A Canadian community perspective

**DOI:** 10.1371/journal.pone.0304457

**Published:** 2024-07-05

**Authors:** Sarah Singh, Shane Goodwin, Shiran Zhong, Abolfazl Avan, Kem Rogers, Vladimir Hachinski, Stephanie Frisbee

**Affiliations:** 1 Robarts Research Institute, University of Western Ontario, London, Ontario, Canada; 2 Department of Sociology, University of Western Ontario, London, Ontario, Canada; 3 Department of Geography, University of Western Ontario, London, Ontario, Canada; 4 Department of Anatomy & Cell Biology, Schulich School of Medicine & Dentistry, University of Western Ontario, London, Ontario, Canada; 5 Department of Clinical Neurological Sciences, and Epidemiology and Biostatistics, Schulich School of Medicine & Dentistry, University of Western Ontario, London, Ontario, Canada; 6 Department of Pathology & Laboratory Medicine, and Epidemiology & Biostatistics, Schulich School of Medicine & Dentistry, University of Western Ontario, London, Ontario, Canada; University of Otago, NEW ZEALAND

## Abstract

**Background:**

Reducing health inequalities among older adults is crucial to ensuring healthy aging is within reach for all. The current study provides a timely update on demographic- and geographic-related inequalities in healthy aging among older adults residing in Canadian communities.

**Methods:**

Data was extracted from the Canadian Health Survey on Seniors [2019–2020] for ~6 million adults aged 65 years and older residing in 10 provinces of Canada. Healthy aging was defined by two indices: 1] health-related quality of life and 2] functional health. Poisson regression models and spatial mapping were used to demonstrate inequalities among age, race, and sex categories, and health regions.

**Results:**

Approximately 90.3% of individuals reported less than perfect quality of life and 18.8% reported less than perfect functional health. The prevalence of less than perfect quality of life was higher for females [PR 1.14, 95% CI;1.02–1.29] and for older adults aged ≥80 years as compared to males and older adults aged ≤79 years [PR 1.66, 95% CI;1.49–1.85]. Similarly, the prevalence of less than perfect functional health was higher for females [PR 1.58, 95% CI;1.32–1.89] and for older adults aged ≥80 years [PR 2.71, 95% CI;2.59–2.84]. Spatial mapping showed that regions of lower quality of life were concentrated in the Prairies and Western Ontario, whereas regions of higher quality of life were concentrated in Quebec.

**Conclusions:**

Amongst older individuals residing in Canadian communities, less than perfect quality of life and functional health is unequally distributed among females, older adults aged ≥80 years, and those residing in the Prairie regions specifically. Newer policy should focus on interventions targeted at these subpopulations to ensure that healthy aging in within reach for all Canadians.

## Introduction

In 2023, Canada recorded its greatest annual population growth in history, however, governing authorities struggle to meet the needs of an aging population [1]. Currently, more than 6 million older adults reside in Canada, with projections that 1 in 4 Canadians will be older than 65 years by 2035 [2]. With increasing costs in medical and long-term care, government and health authorities need to work together to support healthy living into older years [3]. Therefore, successful aging through health promotion and disease prevention in adults aged 65 years and older is top priority.

### Healthy aging

Leading gerontologists Rowe and Kahn in 1987 described successful aging as a multidimensional concept consisting of “high physical, psychological, and social functioning in old age without major diseases” [4]. Research to date has shown that successful aging is strongly influenced by psychosocial factors, multiple lifestyle choices and behaviors such as physical activity, diet and social support [5–8]. In the HYVET [Hypertension in the Very Elderly Trial] and Trial of Non-Pharmacologic Interventions in Elderly [TONE] trials, researchers found that it was possible to achieve target blood pressure in older adults through treatment and diet, and that achieving target blood pressure was associated with reduced the risk of all-cause mortality, fatal stroke, and heart failure [9, 10].

Furthermore, there is a growing need to focus on aging into wellness at home; remaining in the community where families, neighborhoods and religious organizations can provide social support. Innovative research in Europe confirms that the deinstitutionalization of long-term care for older individuals can improve quality of life and increase sustainability of healthcare systems [10]. However, a recent public health report indicates that, while more than 90% of Canadian older adults reside in private dwellings within their communities, more than 30% reported having at least two chronic diseases that may lead to eventual hospitalization or premature mortality [2].

### Measuring healthy aging

Following the declaration of The United Nations labeling 2021–2030 as the “Decade of Healthy Ageing”, there has been a push to measure healthy aging beyond life expectancy and towards measures such as functional health, mental health, cognition, quality of life, overall health and function, and “freedom” from confinement [11]. Such measures encompass positive domains representing health and wellbeing, as opposed to solely negative domains representing morbidity.

The World Health Organization [WHO] defines health related quality of life [HRQOL] as a multidimensional concept that reflects “individuals’ perception of their mental or physical health in the context of the culture and value systems” [12]. Studies indicate that poor HRQOL in individuals aged 65 years and older has been linked to high chronic disease burden, low ability in activities of daily living, and depression [13, 14]. Very few in Canada have identified differences in HRQOL among those aged 65 years and older. In a Canadian study conducted using national data from 1994, authors found a lower HRQOL in those who were older and of lower education and income groups, however, the effect of race was not investigated [15].

Functional health involves the maintenance of functional ability to promote well-being into older years. The premature loss of functional ability, usually due to chronic illness, hinders the process of health aging. Studies show that physical and cognitive interventions including daily exercise and cognitive training can slow the decline in functional ability associated with aging and illness [16]. As with HRQOL, there exists a paucity in Canadian studies, however, recent studies conducted in Sweden, Brazil, China and India have confirmed low income and education as the strongest predictors of poor functional health older adults [17–19].

### Inequalities in healthy aging

Despite the universal health care system in Canada, there remains significant differences in outcomes for healthy aging among population subgroups based on social determinants of health, such as income and education [20, 21] Newer studies have suggested that policies addressing inequalities in aging adopt a two-pronged approach including the reduction of social inequalities at the systems level, as well as, a targeted approach within more susceptible population subgroups at the community level [22–24]. To facilitate the latter, research should be expanded towards identifying those population subgroups most susceptible to poor aging outcomes. The primary objective of this study is to provide a timely update on demographic- and geographic-related inequalities in healthy aging among adults aged 65 years and older residing in Canadian communities from 2019–2020. The secondary objective of this study is to determine whether identified demographic- and geographic-related inequalities persist after accounting for social determinants of health in the study population.

## Methods

The current study uses nationally representative, cross-sectional data from the Canadian Health Survey on Seniors [2019–2020] to examine age-, sex-, race- and regional-based inequalities in health-related quality of life and functional health in adults aged 65 years and older residing in Canadian communities.

### Study sample and data source

The Canadian Health Survey on Seniors [CHSS] is a national cross-sectional survey that utilizes multistage sampling to facilitate national estimates on self-reported health data. The CHSS is derived from the Canadian Community Health Survey—Annual component respondents from ten provinces who are at least 65 years old, along with an oversample in all provinces except Ontario and Quebec. The CHSS data excludes individuals living on reserves and other Aboriginal settlements in the provinces, full-time members of the Canadian Forces, the institutionalized population, and persons living in the Quebec health regions of Région du Nunavik and Région des Terres-Cries-de-la-Baie-James. All individuals in the CHSS 2019–2020 file were included in this study.

### Ethics approval and consent to participate

The Tri-Council Policy Statement [TCPS2]: Ethical Conduct for Research Involving Humans describes five exemption categories: publicly available information [TCPS2 Article 2.2], naturalistic observation [TCPS2 Article 2.3], secondary use of anonymous information [TCPS2 Article 2.4], quality assurance/quality improvement/program evaluation [TCPS2 Article 2.5], and creative practice [TCPS2 Article 2.6]. The Canadian Health Survey on Seniors data, accessed for this study through the Research Data Center at the University of Western Ontario, is an anonymized secondary data source and so qualifies as exempt from REB review by the University of Western Ontario Research Ethics Board in accordance with TCPS2 Article 2.4. All research conducted in reference to this study was performed in accordance with the Declaration of Helsinki.

### Study outcomes

The study outcomes were health-related quality of life [HRQOL] measured by the Health Utilities Index [HUI] and functional health measured by Instrumental and Basic Activities of Daily Living [ADL] Scale.

#### Health Utilities Index [HUI]

The study assessed HRQOL using the Health Utilities Index-3 [HUI3], development and validation of the HUI3 score has been described elsewhere [25, 26]. Briefly, the HUI is a weighted summary preference score based on responses to a multi attribute questionnaire for vision, hearing, speech, mobility, dexterity, emotion, cognition and pain. The function of each attribute is ranked among 5 or 6 levels as shown in S1 Table. General HUI3 scores range from -0.36 to 1.00, with 1.00 indicating perfect health, 0.00 indicating death, and less than 0.00 indicating states worse than death. The term “worse than death” is standard for HUI3 description and represent a severe burden of illness with a substantial deterioration in quality of life. For the purposes of this study, HUI attributes were dichotomized as rank 5 or 6 [perfect health] or ranks less than 5 or 6 [less than perfect health]. Additionally, HUI scores were dichotomized as 1.00 [“perfect quality of life”] or less than 1.00 [“less than perfect quality of life”]. Of note, researchers suggest that 0.01 and 0.03 changes in score represent meaningful differences in HRQOL, however, there exists no consensus on HUI cut points for categorization purposes in population studies [27].

#### Instrumental and Basic Activities of Daily Living [ADL]

The study assessed functional health using the OARS [Older Americans Resources and Services] Multidimensional Functional Assessment Questionnaire [28]. The scale measures the ability to independently perform the following activities: use the phone, go places, go shopping, cook meals, do housework, take medicine, walk, bathe and use the toilet. The scores range from 0 [Excellent/Good] to 5 [Total Impairment] with higher values indicating greater functional impairment. The CHSS included the raw score as well as categories of the overall score as follows: no functional impairment, mild impairment, moderate impairment, severe impairment, total impairment. For the purposes of this study, ADL was dichotomized as “perfect functional health” represented by no functional impairment or “less than perfect functional health” represent by mild to total impairment.

### Study covariates

The study covariates were demographic factors, socioeconomic factors, lifestyle and behavioral factors, and chronic diseases available from CHSS data.

**Demographics.** Age was self-reported age in years. Sex was classified as assigned sex at birth, male or female. Race was classified as White, South Asian, Chinese, Black, Filipino, Latin American, Arab, Southeast Asian, West Asian, Korean, Japanese, Visible minority not otherwise listed and Multiple visible minorities. Immigrant status was classified as yes, immigrant or no, not immigrant to Canada. Martial status was classified as married, living common-law, widowed, separated, divorced, single. Living arrangement was classified as unattached individual living alone, unattached individual living with others, individual living with spouse / partner, parent living with spouse / partner and children, single parent living with children, child living with a single parent, child living with a single parent and siblings, child living with two parents or other. Education was classified as less than secondary, secondary and post-secondary education. Total household income was classified into groups ranging from $0–19,999 to at or above $150,000 CAD.

#### Lifestyle and behaviors

Main weekly activity was classified as working at a paid job or business, vacation, looking for paid work, going to school, caring for children, household work, retired, long-term illness, volunteering, care-giving other than for children. Sense of community belonging is classified as somewhat strong to very weak. Satisfaction with life was classified as very satisfied to very dissatisfied. Body mass index was classified as underweight to obese class III. Diet was described by the frequency of fruit and vegetable consumption classified as less than 5 times to more than 10 times per day. Access to primary care was described by whether the respondent has a regular primary care provider. Smoking was classified as yes, smoked 100 or more cigarettes in lifetime or no, smoked less than 100 cigarettes in lifetime. Alcohol consumption was classified as regular drinker, occasional drinker or did not drink in the past 12 months: regular drinker, occasional drinker, and non-drinker.

#### Chronic diseases

Chronic diseases were classified as yes, no, don’t know or refuse to answer based on the following self reported conditions diagnosed by a clinician: high blood pressure, high blood cholesterol, heart disease, stroke, diabetes, dementia, mood and anxiety disorders.

### Statistical analysis

Descriptive analyses were conducted for the study outcome and covariates to describe the study population. Mean values were calculated for continuous variables, frequency and proportions were calculated for categorical variables.

Based on the structured data collection, missing data comprised less than 10% of study data and missingness was believed to be no different from random, therefore no imputation techniques were employed. Missing data were reported as ‘not stated’ or ‘refused to answer’ in the survey as described Table 1. Individuals missing data on study outcomes were excluded from model analyses. Individuals missing data on study covariates were included in model analyses and coded as a separate ‘missing’ category or value within each covariate.

**Table 1:** Characteristics of the weighted study population of adults aged 65 years and older residing in Canadian communities, Canadian Healthy Survey on Seniors 2019–2020 [n = 6,437,939].

Characteristics		Frequency [%] or Mean [s.d]
**Outcomes**		
Health Utilities Index [HUI]^a^	HUI score = 1.00 [perfect quality of life]	588712 [9.1]
	HUI score < 1.00 [less than perfect quality of life]	5510728 [85.6]
	Missing [not stated]	338498 [5.3]
Activities of Daily Living^b^	No impairment [perfect functional health]	5114340 [79.4]
	At least some impairment [less than perfect functional health]	1180849 [18.4]
	Missing [not stated]	142749 [2.2]
**Demographics**		
Average age [years]		74.2 [0.07]
Sex	Male	2995382 [46.5]
	Female	3442557 [53.5]
Race/Ethnicity	South Asian	172784 [2.7]
	Chinese	188908 [2.9]
	Black	102601 [1.6]
	Filipino	55878 [0.9]
	Latin American	36423 [0.6]
	Arab	33549 [0.5]
	Southeast Asian	39156 [0.6]
	West Asian	22207 [0.3]
	Korean	12906 [0.2]
	Japanese	18750 [0.3]
	Visible minority	12571 [0.2]
	Multiple visible minorities	20148 [0.3]
	Not a visible minority	5670349 [88.1]
	Not stated	51707 [0.8]
Marital status	Married	3745625 [58.2]
	Living common-law	342561 [5.3]
	Widowed	1215218 [18.9]
	Separated	134287 [2.1]
	Divorced	556585 [8.6]
	Single, never married	433583 [6.7]
	Don’t know	1526 [0.02]
	Refusal	8553 [0.13]
Education	Less than secondary school graduation	1342153 [20.8]
	Secondary school graduation, no post-secondary education	1438918 [22.3]
	Post-secondary certificate diploma or univ degree	3507024 [54.5]
	Not stated	149843 [2.3]
Immigrant status	Immigrant	1755041 [27.3]
	Not immigrant	4674367 [72.6]
	Refusal	8531 [0.1]
Total household income	No income or income loss	7370 [0.1]
	Less than $5,000	5926 [0.09]
	$5,000 to $9,999	12565 [0.2]
	$10,000 to $14,999	34120 [0.5]
	$15,000 to $19,999	206447 [3.2]
	$20,000 to $29,999	601059 [9.3]
	$30,000 to $39,999	755949 [11.7]
	$40,000 to $49,999	634596 [9.9]
	$50,000 to $59,999	564107 [8.8]
	$60,000 to $69,999	516987 [8.0]
	$70,000 to $79,999	459272 [7.1]
	$80,000 to $89,999	395584 [6.1]
	$90,000 to $99,999	363966 [5.6]
	$100,000 to $149,999	1019062 [15.8]
	$150,000 or more	860929 [13.4]
Living arrangement	Unattached individual living alone	1842088 [28.6]
	Unattached individual living with others	184414 [2.9]
	Individual living with spouse / partner	3425698 [53.2]
	Parent living with spouse / partner and children	340786 [5.3]
	Single parent living with children	198996 [3.1]
	Child living with a single parent	17650 [0.3]
	Child living with a single parent and siblings	1796 [0.03]
	Child living with two parents or other	425002 [6.6]
	Not stated	1509 [0.02]
**Lifestyle and Behaviors**		
Main weekly activity	Working at a paid job or business	666948 [10.4]
	Vacation [from paid work]	17419 [0.3]
	Looking for paid work	19997 [0.3]
	Going to school [including vacation from school]	969 [0.02]
	Caring for children	19049 [0.3]
	Household work	122838 [1.9]
	Retired	5383313 [83.6]
	Long-term illness	78425 [1.2]
	Volunteering	37826 [0.6]
	Care-giving other than for children	13087 [0.2]
	Other	61663 [0.9]
	Don’t know	1706 [0.03]
	Refusal	386 [0.006]
	Not stated	14311 [0.2]
Sense of belonging to local community	Very strong	1489454 [23.1]
	Somewhat strong	2943033 [45.7]
	Somewhat weak	1053178 [16.4]
	Very weak	375185 [5.8]
	Don’t know	96651 [1.5]
	Refusal	2897 [0.05]
	Not stated	477541 [7.4]
Satisfaction with life in general	Very Satisfied	2513195 [39.0]
	Satisfied	2840781 [44.1]
	Neither satisfied nor dissatisfied	363326 [5.6]
	Dissatisfied	137322 [2.1]
	Very Dissatisfied	33315 [0.5]
	Not stated	549999 [8.5]
Body Mass Index	Underweight	77325 [1.2]
	Normal weight	1855406 [28.8]
	Overweight	2239905 [34.8]
	Obese—Class I	1172748 [18.2]
	Obese—Class II	341023 [5.3]
	Obese—Class III	133556 [2.1]
	Don’t know	4047 [0.06]
	Refusal	613929 [9.5]
Smoked more than 100 cigarettes	Yes	3250201 [50.5]
	No	3162259 [49.1]
	Don’t know	23240 [0.4]
	Refusal	2238 [0.03]
Alcohol consumption	Regular drinker	3426974 [53.2]
	Occasional drinker	1061551 [16.5]
	Did not drink in the last 12 months	1921426 [29.8]
	Not stated	27987 [0.4]
Fruit and vegetable consumption	Eats fruits and vegetables less than 5 times per day	2379070 [37.0]
	Eats fruits and vegetables between 5 and 10 times per day	917068 [14.2]
	Eats fruits and vegetables more than 10 times per day	61047 [0.9]
	Valid skip	2702084 [42.0]
	Not stated	378669 [5.9]
Has a regular provider	Yes	6038600 [93.8]
	No	390819 [6.1]
	Don’t know	7134 [0.1]
	Refusal	1385 [0.02]
**Chronic diseases**		
Chronic disease	Has at least one chronic condition	573974 [89.2]
	Has no chronic conditions	66104 [10.3]
	Not stated	37111 [0.6]
Has high blood pressure	Yes	2810679 [43.7]
	No	3601821 [55.9]
	Don’t know	24302 [0.4]
	Refusal	1136 [0.02]
Has high blood cholesterol / lipids	Yes	1855925 [28.8]
	No	4494279 [69.8]
	Don’t know	83319 [1.3]
	Refusal	4416 [0.07]
Has heart disease	Yes	973323 [15.1]
	No	5418400 [84.2]
	Don’t know	44993 [0.7]
	Refusal	1222 [0.02]
Suffers from the effects of a stroke	Yes	262870 [4.1]
	No	6165648 [95.8]
	Don’t know	8806 [0.1]
	Refusal	614 [0.01]
Has diabetes	Yes	1217242 [18.9]
	No	5211340 [80.9]
	Don’t know	8789 [0.1]
	Refusal	566 [0.01]
Has Alzheimer’s Disease or any other dementia	Yes	140388 [2.2]
	No	6285885 [97.6]
	Don’t know	11173 [0.02]
	Refusal	492 [0.01]
Has a mood disorder [depression, bipolar, mania, dysthymia]	Yes	414350 [6.4]
	No	6015687 [93.4]
	Don’t know	7040 [0.11]
	Refusal	862 [0.01]
Has an anxiety disorder [phobia, OCD, panic]	Yes	362338 [5.6]
	No	6066991 [94.2]
	Don’t know	7498 [0.11]
	Refusal	1111 [0.02]

^a^ Measured by Health Utilities Index 3 tool

^b^ Measured by the OARS Activities of Daily Living Scale

Prevalence ratios, obtained by Poisson regression models with robust variance, were used to assess demographic inequalities based on age, sex, and race. Numerous studies have confirmed that the estimation of prevalence ratios in cross sectional studies to assess differences is more appropriate than the estimation of odds ratios [29, 30]. In order to assess the impact of social determinants on prevalence ratio estimates, both crude [unadjusted] and adjusted models were derived. Models were adjusted for all study covariates listed in Table 1.

For ease of interpretation, age, race and sex were dichotomized in Poisson models, where one group was the reference, and the other group was the comparator. The reference group represented the proportional majority in the study population. For age, adults aged 65–79 years were the reference group and adults aged 80 years and older were the comparator group. For sex assigned at birth, males were the reference group and females were the comparator group. For race, respondents identifying as White were the reference group and respondents identifying as all other racial groups except White were the comparator group. The term “all other racial groups except White” was recommended for use by the American Heart Association Structural Racism and Health Equity Language Guide [31]. Of note, each unique race that comprised all other racial groups except White was listed and included in descriptive analyses of the study population [Table 1].

The statistical analysis was performed using SAS version 9.4 [SAS Institute, Cary, NC, USA]. All estimates and confidence intervals were derived using the sampling weights provided by the Canadian Health Survey on Seniors [CHSS] to account for the complex, multistage sampling design and obtain precise estimates of variation.

For the final stage of analysis, geographic inequalities were assessed using spatial analysis in R software. The geographic unit of analysis was Health Regions [HRs], legislated administrative areas that represent geographic areas of responsibility for hospital boards or regional health authorities [32]. There were 167 HRs in Canada from 2019–2020, with Territories excluded. For simplicity in mapping, spatial autocorrelation for the average Health Utilities Index Score aggregated to the HR level was assessed using Moran’s I statistic. HRs were categorised based on quartile of average Health Utilities Index Score. HRs were expressed in a row-standardized spatial weights matrix, defining neighbouring regions using queen’s-based contiguity. Monte Carlo simulations with 1000 random permutations were used to test statistical significance of Moran’s I at p<0.05. In the presence of statistically significant clustering, HRs that have a larger spatial autocorrelation relative to other regions, Local Indicators of Spatial Association [LISA] were estimated. All data files used to create maps were publicly available from the data holdings and used in compliance with the Statistics Canada Open License Agreement [33, 34].

## Results

Table 1 describes the characteristics of the study population including a weighted total of 6,437,939 individuals aged 65 years and older residing in Canadian households in 2019–2020. Overall, the average age of the study population was 74.2 years. Approximately half of individuals were female [53.5%], married [58.2%] and educated past secondary school [54.5%]. Most individuals were White [88.1%] with at least one chronic condition [89.2%]. There were 1,180,849 [18.4%] individuals with less than perfect functional health and 5,510,728 [85.6%] individuals with less than perfect quality of life [HUI<1.00].

**Fig 1:**
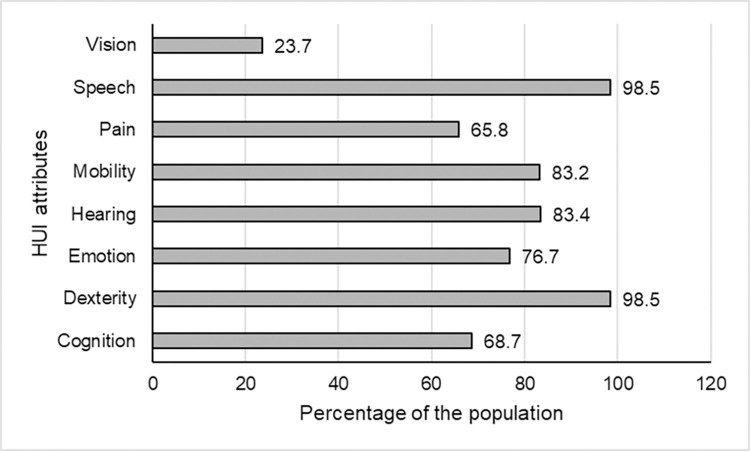
The weighted proportion of adults aged 65 years and older residing in Canada with perfect health in 8 Health Utility Index attributes, Canada Healthy Survey on Seniors 2019–2020. HUI attributes include: cognition, dexterity, emotion, hearing, mobility, pain, speech, and vision. There are 6–7 categories within each HUI attribute, with category 1 [graphed] representing perfect health and remaining categories representing declining health. Further description of HUI attributes and categories are shown in S1 Table.

Fig 1 demonstrates the proportion of individuals within categories of the eight HUI attributes: vision, hearing, speech, mobility, dexterity, emotion, cognition and pain. Approximately 68.7% had perfect health in cognition, 98.5% had perfect health in dexterity, 76.7% had perfect health in emotion, 83.4% had perfect health in hearing, 83.2% had perfect health in mobility, 65.8% had perfect health in pain, 98.5% had perfect health in speech, and 23.7% had perfect health in vision.

**Fig 2:**
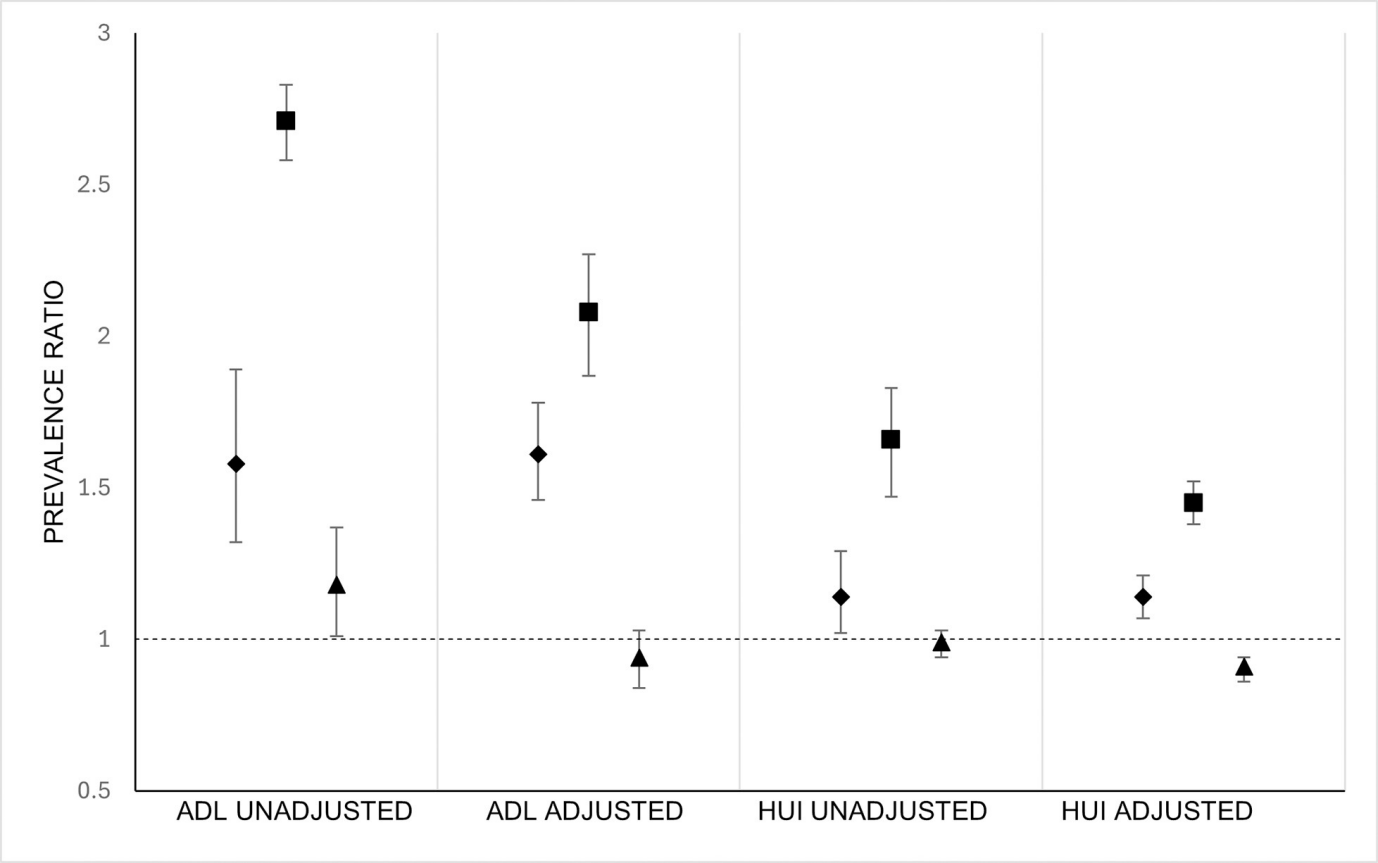
Prevalence ratios for less than perfect health [Health Utilities Index] and less than perfect functional health [Activities of Daily Living] in adults aged 65 years and older residing in Canadian communities, Canadian Healthy Survey on Seniors 2019–2020. HUI: Health Utilities Index [modeled as less than perfect health-related quality of life], ADL: activities of daily living [modeled as less than perfect functional health], unadjusted: crude models not including study covariates, adjusted: models including study covariates, Legend: circle- female vs. male, square- 79 years and younger vs 80 years and older, diamond- all other vs White race.

Prevalence ratios for less than perfect health are shown in Fig 2. The prevalence of less than perfect HRQOL was higher for females as compared to males [PR 1.14, 95% CI;1.02–1.29] and for adults 80 years and older as compared to adults 79 years and younger [PR 1.66, 95% CI;1.49–1.85] only. Significant differences in race were not noted for less than perfect HRQOL. The prevalence of less than perfect functional health was higher for females [PR 1.58, 95% CI;1.32–1.89] and for adults 80 years and older [PR 2.71, 95% CI;2.59–2.84]. The prevalence of less than perfect functional health was marginally higher for all other racial groups except White compared to White [PR 1.15, 95% CI;0.99–1.35]. Prevalence ratios were reduced minimally but remained statistically significant after accounting for all social determinants of health in adjusted models.

Prevalence ratios for less than perfect health in each of the eight HUI attributes, accounting for all social determinants of health, are shown in Table 2. Of note, the prevalence of poor hearing and mobility were more than doubled in the adults 80 years and older; [PR 2.03, 95% CI;1.81–2.27] and [PR 2.14, 95% CI;2.04–2.24] respectively. The prevalence of poor mobility and pain were greater for females; [PR 1.39, 95% CI;1.35–1.43] and [PR 1.29, 95% CI;1.24–1.34] respectively. The prevalence of poor dexterity was approximately 50% higher for all other racial groups except White as compared to White with prevalence ratio [PR 1.44, 95% CI;1.03–1.12] respectively.

**Table 2:** Prevalence ratios for poor health in Health Utilities Index attributes in adults aged 65 years and older residing in Canadian communities, Canadian Healthy Survey on Seniors 2019–2020.

Health Utilities Index Attributes	Adjusted Prevalence ratio^a^	95% CI
**Cognition**		
Male vs female	0.98	0.95–1.01
Younger vs older	1.23	1.19–1.28^b^
White vs all other	0.97	0.85–1.12
**Dexterity**		
Male vs female	1.08	0.64–1.83
Younger vs older	1.16	0.94–1.42
White vs all other	1.44	1.03–1.12^b^
**Emotional**		
Male vs female	0.92	0.82–1.05
Younger vs older	1.06	1.01–1.11^b^
White vs all other	0.93	0.78–1.10
**Hearing**		
Male vs female	0.71	0.61–0.84
Younger vs older	2.03	1.81–2.27^b^
White vs all other	0.67	0.58–0.79
**Mobility**		
Male vs female	1.39	1.35–1.43^b^
Younger vs older	2.14	2.04–2.24^b^
White vs all other	0.78	0.72–0.84
**Pain**		
Male vs female	1.29	1.24–1.34^b^
Younger vs older	1.08	0.97–1.20
White vs all other	0.96	0.81–1.13
**Speech**		
Male vs female	0.57	0.41–0.79
Younger vs older	1.42	1.11–1.83^b^
White vs all other	0.95	0.60–1.49
**Vision**		
Male vs female	1.06	1.05–1.08^b^
Younger vs older	0.98	0.97–0.99
White vs all other	0.97	0.93–1.00

^a^ All eight models conducted independently and adjusted for study covariates.

^b^ Indicates statistical significance [PR does not include 1.00]

**Fig 3:**
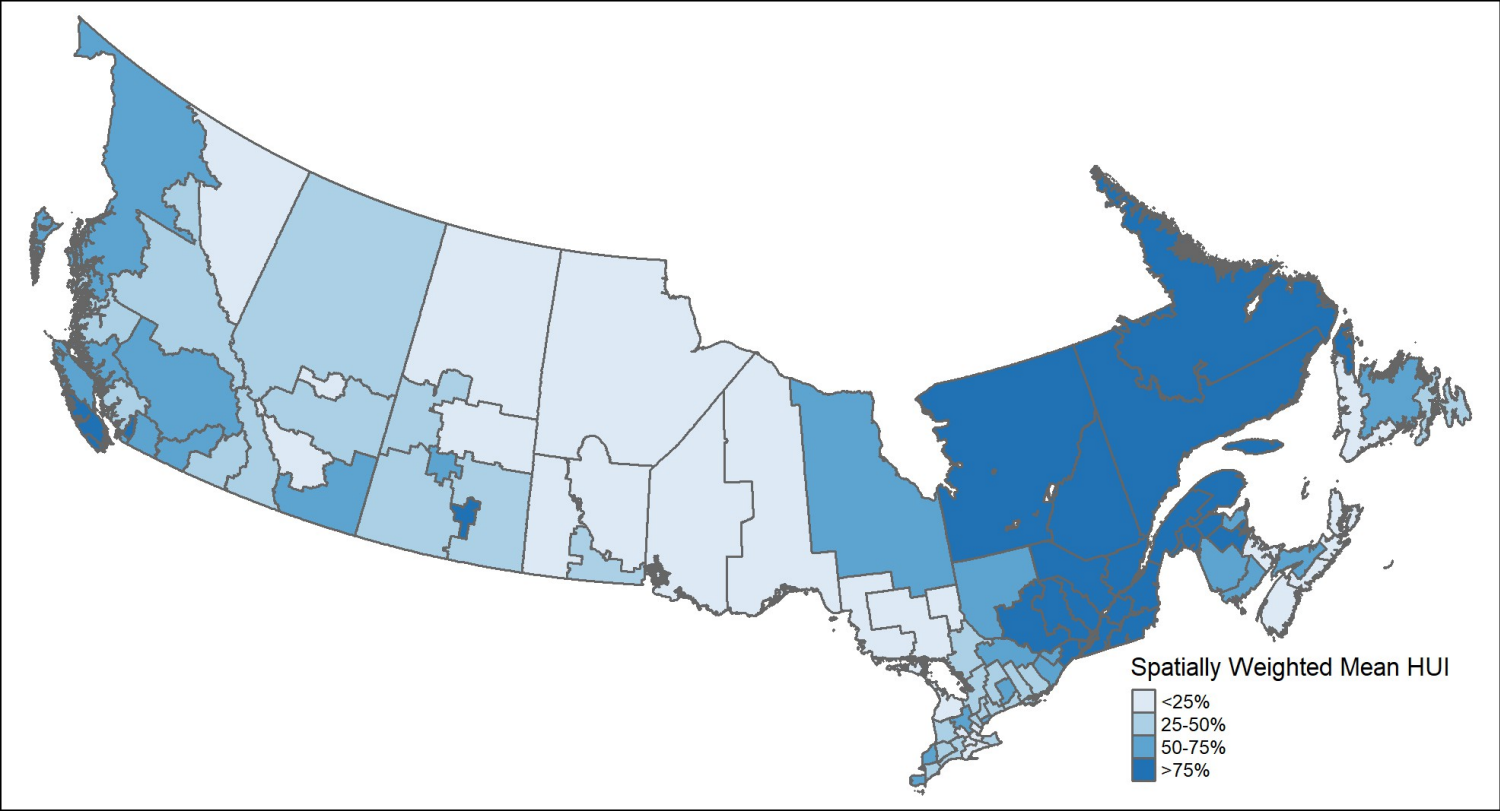
Spatial map of Canada health regions based on average Health Utilities Score, Canada Health Survey on Seniors 2019–2020. Average Health Utilities Scores categorised into quartiles with darker regions representing areas with highest average Health Utilities Score or best quality of life, and lighter regions representing areas with lowest average Health Utilities Score or worst quality of life.

**Fig 4:**
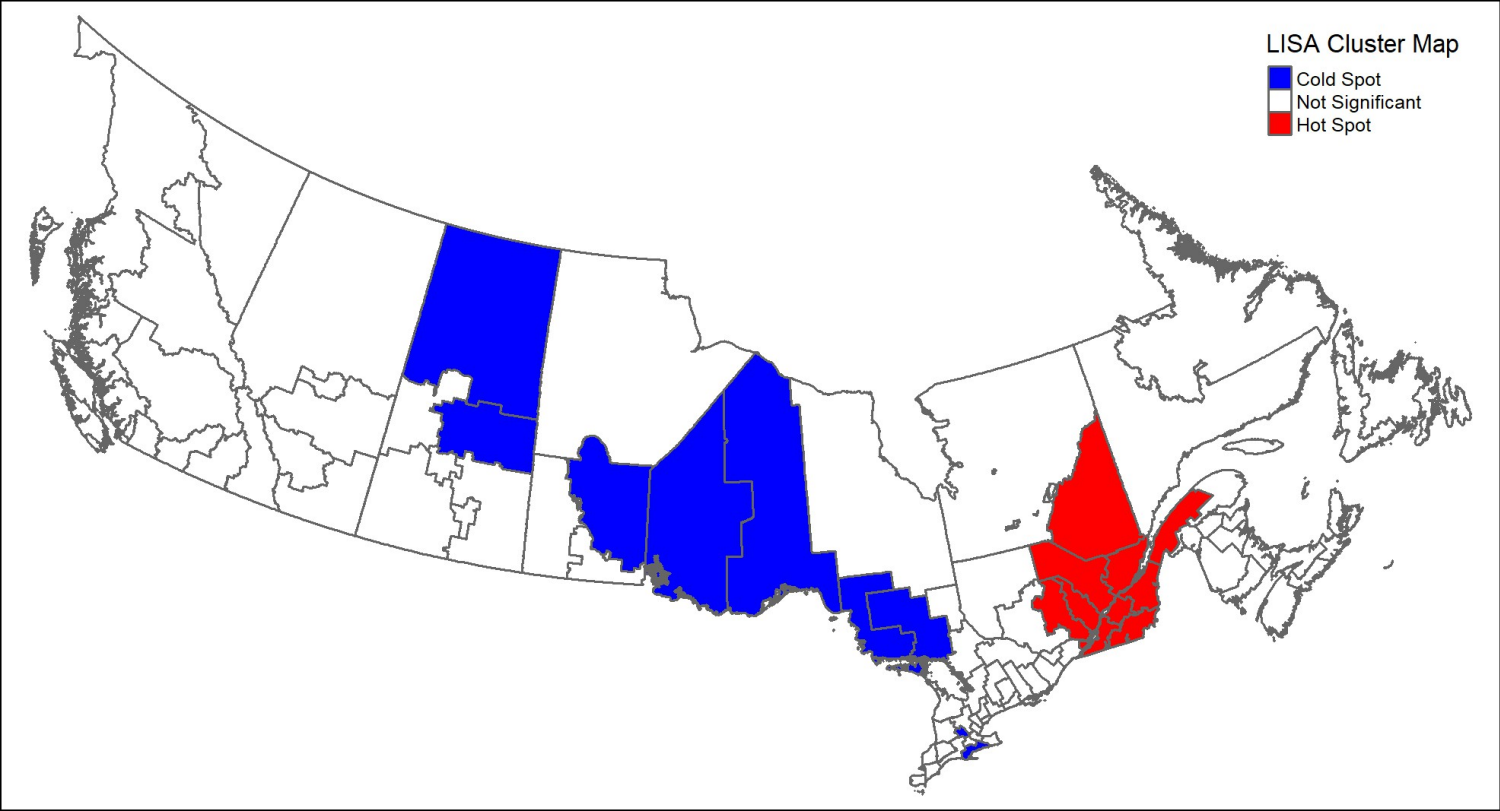
LlSA cluster map of Canada Health Regions based on average Health Utilities Score, Canada Health Survey on Seniors 2019–2020. Based on LISA analyses, Health Regions are divided into cold spots, hot spots, and not significant. Cold spots represent statistically significant regions of lowest Average Health Utilities Scores surrounded by other low scoring regions. Hot spots represent statistically significant regions of highest Average Health Utilities Scores surrounded by other high scoring regions. Not significant spots were not different from the distribution of Average Health Utilities Scores across all regions.

Results of spatial analysis demonstrating regional-based inequalities are shown in Figs 3 and 4. The Global Moran’s I was statistically significant [0.34; p<0.001] suggesting that the spatial distribution of the Health Utilities Index Score among HRs was not random. Spatially weighted maps demonstrated regions of lowest quality of life in the Prairies and Western Ontario, whereas regions of highest quality of life were found in Atlantic Canada and Quebec [Fig 3]. Specifically, LISA cluster maps demonstrated hot spots in Quebec—statistically significant clusters of high scoring HRs that were surrounded by other high scoring regions, and cold spots in Ontario, Manitoba and Saskatchewan—statistically significant clusters of low scoring HRs that were surrounded by other low scoring regions. [Fig 4].

## Discussion

Our study on adults aged 65 years and older residing in Canadian communities revealed significant age- and sex-based inequalities in healthy aging that could not be fully explained by social determinants of health. Additionally, healthy aging is highly spatially dependent with certain regions of Canada experiencing far better quality of life than others. Healthy aging requires policies and interventions that are integrational, that is, addressing social determinants while also targeting those subpopulations most in need of urgent care.

In 2019–2020, females and adults aged 80 years and over experienced a higher prevalence of less than perfect health-related quality of life [HRQOL] and functional health as compared to males and adults aged 79 years and younger, even after accounting for demographics, lifestyle and behavioral choices, and chronic diseases. Specifically, females experienced a higher prevalence of poor mobility and pain, adults aged 80 years and older experienced a higher prevalence of poor cognition, and individuals of all other racial groups except White experienced a higher prevalence of poor dexterity. Furthermore, findings demonstrate statistically significant differences in average HRQOL with regions in Quebec experiencing the highest quality of life in Canada.

Within the past decade, very few studies have examined inequalities in HRQOL in the aging population at the national level. Authors of a Swedish study showed a strong association between poor HRQOL in adults aged 85 years and older, with lower HRQOL in women as compared to men. Lower HRQOL was associated with greater depression and prevalence of chronic disease in the study sample [35]. Similar findings were noted in an Iranian sample where inequalities in HRQOL could not be eliminated after adjusting for sociodemographic characteristics and chronic disease conditions [36]. Our study confirms similar findings and provides timely updates on the Canadian contribution to the literature.

Race-based inequalities in HRQOL have been found in the US, with Black and Hispanic individuals generally experiencing lower HRQOL as compared to White individuals in older populations. Researchers attribute this finding to a life course exposure of vulnerable racial groups to adverse socioeconomic conditions that uniquely impact health in older age [37]. Although our study did not assess life course exposure, while social determinants of health did not eliminate sex- and age-based inequalities, they fully accounted for trending race-based inequalities in HRQOL. Findings posit that social determinants may have a stronger influence on race-based inequalities than sex- or age-based inequalities in HRQOL. Further research is needed to explore possible interactive effects.

Better functional health is strongly associated with higher quality of life, greater independence and reduced healthcare costs in aging individuals [38, 39]. It is important to note that although some decline in functional health is expected with age, even mild functional impairment in ADL doubles the risk of mortality as compared to no functional impairment [40]. Therefore, the higher prevalence of less than perfect functional health in females and adults aged 80 years and older in the Canadian community, not yet institutionalized, is of great concern and warrants further investigation. Although difficult to assess, existing research suggests that differences in ADL between men and women may be attributed to gender roles and expectations within the older generations [41].

Global studies have highlighted socio-economic inequalities in functional health in older adults. In a study conducted in India, authors confirm that education and wealth explained most of the socio-economic inequalities in ADL among older adults [18]. Similar results were noted in recent studies conducted in both China and Brazil, however, equivalent studies in Canada were not found in the literature [17, 42]. Our study noted a higher prevalence of poor functional health in females and adults aged 80 years and older, that could not be accounted for by social determinants of health. Importantly, and undeniably, social determinants of health contribute greatly to inequalities in healthy aging. However, age- and sex- based inequalities persist independently and should be addressed using tailored and targeted interventions.

Findings of this study confirm the spatial distribution of HRQOL among older adults residing in Canadian communities, showing a striking gradient of worsening HRQOL away from coastal regions. Generally, in Canada, coastal regions represent more populous, urban areas while plain or central regions represent more remote, rural areas. A national report observed lower socioeconomic status, characterized by lower education, income and housing costs, in the northern and eastern regions of Canada as compared to the southern and western region [43]. While our study does not investigate a causal link between socioeconomic status and HRQOL, it is important to note the similarities in geographic distribution between the two attributes. Developing regional interventions targeted specifically at such areas may prove to be a more effective method for promoting healthy aging as opposed to a one-size-fits-all national policy that benefits some regions more than others.

Strengths of the study include the use of nationally representative data and validated scales for HRQOL and functional health that can are suitable to monitoring health in older populations. A limitation of the HUI3 measure was the method of classification used in this study. The HUI3 is a generic measure used in a wide range of studies. In other studies, the clinical significance of cut points for HUI3 appear to be based on the context for its use. Many studies tend to avoid this generalizability issue by using disease-specific instruments for HRQOL, however, our study does not refer to a specific disease. Therefore, we have maintained the use of the HUI3, however, we have not relied on any specific cut point. We used the broadest categorization possible based on the most reliable threshold of the measure, i.e. 1.00 indicates perfect health. Nevertheless, we recognize that this dichotomization may lead to classification bias in quantifying HRQOL.

Although representative, another important limitation of the data source was the lack of racial/ethnic diversity within the study population and the exclusion of the Indigenous population. Other limitations of the study include the cross-sectional nature of the data such that inferences could not be drawn based on causality. Further, regional level results were aggregated and thus cannot be used to make individual level conclusions on geographic distributions.

Future studies should build on existing research by identifying and quantifying inequalities in healthy aging, perhaps using a life course approach, examining how inequalities may be inherited and executed from birth to aging.

## Conclusions

Study findings suggest addressing quality of life and functional health measures, such as cognition and pain, particularly among females and older adults, to reduce the burden of disability and maintain independence in community dwelling individuals. Policy makers need to focus on regional level interventions as opposed to a national one-size-fits-all approach that currently benefits some areas of the country more than others. A combination of targeted individual-level interventions and systemic population-based policies are vital to supporting healthy aging and reducing inequalities in the aging population.

## Supporting information

S1 Table Health Utilities Index [HUI3] attributes in the study population of adults aged 65 years and over residing in Canadian communities, Canadian Healthy Survey on Seniors 2019–2020. (DOCX)
