# Supplementary material for: Inequalities in health-related quality of life and functional health of an aging population: A Canadian community perspective
Source: PLoS One. 2024 Jul 5;19(7):e0304457. doi: 10.1371/journal.pone.0304457 (PMC11226017; doi:10.1371/journal.pone.0304457)
Supplement: S1 Table — (DOCX) [file pone.0304457.s001.docx]

| **HUI attributes^a^** | **Level** |  | **Weighted Frequency (%)** |
| --- | --- | --- | --- |
| **Cognition** | 1 | Able to remember most things, think clearly and solve day to day problems. | 4425636 (68.7) |
|  | 2 | Able to remember most things, but have a little difficulty when trying to think and solve day to day problems. | 211923 (3.3) |
|  | 3 | Somewhat forgetful, but able to think clearly and solve day to day problems. | 1204342 (18.7) |
|  | 4 | Somewhat forgetful and have a little difficulty when trying to think or solve day to day problems. | 406169 (6.3) |
|  | 5 | Very forgetful, and have great difficulty when trying to think or solve day to day problems | 133126 (2.1) |
|  | 6 | Unable to remember anything at all, and unable to think or solve day to day problems. | 31279 (0.5) |
|  |  | Cannot assess | 31279 (0.5) |
| **Dexterity** | 1 | Full use of two hands and ten fingers. | 6338496 (98.5) |
|  | 2 | Limitations in the use of hands or fingers, but does not require special tools or help of another person. | 45885 (0.7) |
|  | 3 | Limitations in the use of hands or fingers, is independent with use of special tools (does not require the help of another person). | 3812 (0.06) |
|  | 4 | Limitations in the use of hands or fingers, requires the help of another person for some tasks (not independent even with use of special tools). | 27058 (0.4) |
|  | 5 | Limitations in use of hands or fingers, requires the help of another person for most tasks (not independent even with use of special tools). | 13172 (0.2) |
|  | 6 | Limitations in use of hands or fingers, requires the help of another person for all tasks (not independent even with use of special tools). | 3374 (0.05) |
|  |  | Cannot assess | 6142 (0.1) |
| **Emotion** | 1 | Happy and interested in life. | 4935469 (76.7) |
|  | 2 | Somewhat happy. | 1272373 (19.8) |
|  | 3 | Somewhat unhappy. | 151670 (2.3) |
|  | 4 | Very unhappy. | 41724 (0.6) |
|  | 5 | So unhappy that life is not worthwhile. | 10780 (0.2) |
|  |  | Cannot assess | 25922 (0.4) |
| **Hearing** | 1 | Able to hear what is said in a group conversation with at least three other people, without a hearing aid. | 5369964 (83.4) |
|  | 2 | Able to hear what is said in a conversation with one other person in a quiet room without a hearing aid, but requires a hearing aid to hear what is said in a group conversation with at least three other people. | 480553 (7.5) |
|  | 3 | Able to hear what is said in a conversation with one other person in a quiet room with a hearing aid, and able to hear what is said in a group conversation with at least three other people, with a hearing aid. | 173939 (2.7) |
|  | 4 | Able to hear what is said in a conversation with one other person in a quiet room, without a hearing aid, but unable to hear what is said in a group conversation with at least three other people even with a hearing aid. | 172618 (2.7) |
|  | 5 | Able to hear what is said in a conversation with one other person in a quiet room with a hearing aid, but unable to hear what is said in a group conversation with at least three other people even with a hearing aid. | 46821 (0.7) |
|  | 6 | Unable to hear at all. | 30341 (0.5) |
|  |  | Cannot assess | 163702 (2.5) |
| **Mobility** | 1 | Able to walk around the neighbourhood without difficulty, and without walking equipment. | 5354181 (83.2) |
|  | 2 | Able to walk around the neighbourhood with difficulty; but does not require walking equipment or the help of another person. | 159465 (2.5) |
|  | 3 | Able to walk around the neighbourhood with walking equipment, but without the help of another person. | 636261 (9.9) |
|  | 4 | Able to walk only short distances with walking equipment, and requires a wheelchair to get around the neighbourhood. | 35191 (0.5) |
|  | 5 | Unable to walk alone, even with walking equipment. Able to walk short distances with the help of another person, and requires a wheelchair to get around the neighbourhood. | 165092 (2.6) |
|  | 6 | Cannot walk at all. | 66085 (1.0) |
|  |  | Cannot assess | 21663 (0.3) |
| **Pain** | 1 | Free of pain and discomfort. | 4237722 (65.8) |
|  | 2 | Mild to moderate pain that prevents no activities. | 653986 (10.1) |
|  | 3 | Moderate pain that prevents a few activities. | 677737 (10.5) |
|  | 4 | Moderate to severe pain that prevents some activities. | 481637 (7.5) |
|  | 5 | Severe pain that prevents most activities. | 338767 (5.3) |
|  |  | Cannot assess | 48090 (0.7) |
| **Speech** | 1 | Able to be understood completely when speaking with strangers or friends. | 6344537 (98.5) |
|  | 2 | Able to be understood partially when speaking with strangers but able to be understood completely when speaking with people who know me well. | 43613 (0.7) |
|  | 3 | Able to be understood partially when speaking with strangers or people who know me well. | 8900 (0.1) |
|  | 4 | Unable to be understood when speaking with strangers but able to be understood partially by people who know me well. | 20687 (0.3) |
|  | 5 | Unable to be understood when speaking to other people (or unable to speak at all). | 5214 (0.08) |
|  |  | Cannot assess | 14988 (0.2) |
| **Vision** | 1 | Able to see well enough to read ordinary newsprint and recognize a friend on the other side of the street, without glasses or contact lenses. | 1528981 (23.7) |
|  | 2 | Able to see well enough to read ordinary newsprint and recognize a friend on the other side of the street, but with glasses. | 4627436 (71.9) |
|  | 3 | Able to read ordinary newsprint with or without glasses but unable to recognize a friend on the other side of the street, even with glasses. | 60422 (0.9) |
|  | 4 | Able to recognize a friend on the other side of the street with or without glasses but unable to read ordinary newsprint, even with glasses. | 70387 (1.1) |
|  | 5 | Unable to read ordinary newsprint and unable to recognize a friend on the other side of the street, even with glasses. | 50375 (0.8) |
|  | 6 | Unable to see at all. | 8343 (0.1) |
|  |  | Cannot assess | 91995 (1.4) |

^a^ Health Utilities Index consists of eight individual attributes, each describing 6-7 levels of functioning.
